# High Performance of Histidine-Rich Protein 2 Based Rapid Diagnostic Tests in French Guiana are Explained by the Absence of *pfhrp2* Gene Deletion in *P. falciparum*

**DOI:** 10.1371/journal.pone.0074269

**Published:** 2013-09-23

**Authors:** Mélanie Trouvay, Georges Palazon, Franck Berger, Béatrice Volney, Denis Blanchet, Emilie Faway, Damien Donato, Eric Legrand, Bernard Carme, Lise Musset

**Affiliations:** 1 Laboratoire de parasitologie, Centre National de Référence de la Chimiorésistance du Paludisme, région Antilles-Guyane, Institut Pasteur de la Guyane, Cayenne, French Guiana, France; 2 Laboratoire Hospitalo-Universitaire de Parasitologie et Mycologie Médicale, Equipe Accueil 3593, Unité de Formation et de Recherche de Médecine de l’Université des Antilles et de la Guyane, Cayenne, French Guiana, France; 3 Unité d’épidémiologie, Institut Pasteur de la Guyane, Cayenne, French Guiana, France; Quensland University of Technology, Australia

## Abstract

**Background:**

Care for malaria patients in endemic areas has been improved through the increasing use of Rapid Diagnostic Tests (RDTs). Most RDTs target the histidine-rich protein-2 antigen (PfHRP_2_) to detect *P. falciparum*, as it is abundant and shows great heat stability. However, their use in South America has been widely questioned following a recent publication that pinpoints the high prevalence of Peruvian field isolates lacking the gene encoding this protein. In the remote rural health centers of French Guiana, RDTs are the main diagnosis tools. Therefore, a study of PfHRP2 RDT performances and *pfhrp2* genotyping was conducted to determine whether a replacement of the current pLDH-based kit could be considered.

**Methods:**

The performance study compared the SD Malaria Ag test P.f/Pan® kit with the current gold standard diagnosis by microscopy. The prevalence of *pfhrp2* and *pfhrp3* deletions were evaluated from 221 *P. falciparum* isolates collected between 2009 and 2011 in French Guiana.

**Results:**

Between January 2010 and August 2011, 960 suspected cases of malaria were analyzed using microscopy and RDTs. The sensitivity of the SD Malaria Ag test P.f/Pan® for detection of *P. falciparum* was 96.8% (95% CI: 90.9–99.3), and 86.0% (95% CI: 78.9–91.5) for the detection of *P. vivax*. No isolates (95% CI: 0–4.5) lacking either exon of the *pfhrp_2_* gene were identified among the 221 *P. falciparum* isolates analyzed, but 7.4% (95% CI: 2.8–15.4) lacked the exon 2 part of the *pfhrp_3_* gene.

**Conclusions:**

Field isolates lacking either exon of the *pfhrp_2_* gene are absent in this western part of South America. Despite its sensibility to detect *P. vivax*, the SD Malaria Ag test P.f/Pan® kit is a satisfying alternative to microscopy in remote health centers, where it is difficult to provide highly skilled microscopists and to maintain the necessary equipment.

## Introduction

In the late 1990’s, the introduction of Rapid Diagnosis Tests (RDT) for malaria provided a rapid, accurate and accessible diagnostic tool that reduced clinical diagnosis in endemic areas where skills and materials are lacking. As malaria symptoms are non-specific, this lowered presumptive treatment and therefore reduced drug pressure on the parasite population. Numerous commercial kits exist. WHO therefore evaluated the performances of a wide panel of commercial kits, using a standardized procedure in order to compare performances between them. The results of these evaluations have been published and included in general guidance for health workers [1]–[4]. The selection of an appropriate RDT in a given region is based on the epidemiology of malaria and the performances of the kit in that particular area [5].

French Guiana is an outermost region of France located in Amazonia between North Brazil and Suriname. The health system in this region consists of a network of medical structures that allow patients to be treated close to their homes. The Hospital of Cayenne, the main town of French Guiana, administers these rural health-posts, which are spread over the entire territory. In the smallest of these healthcare facilities, the health provider team is composed of just one nurse. In this Amazonian area, malaria is endemic. In 2011, *P. falciparum* and *P. vivax* represented 31% and 68.5% of the 1,205 cases, respectively [6]. *P. malariae* cases are occasional. Transmission occurs along thousands of miles of rivers with a low number of focal points in the coastal area where the majority (90%) of the population lives. This transmission is closely linked with the mostly illegal gold mining activities [7], [8]. In this context, the containment of the disease in these areas is difficult. Until 2000, malaria diagnosis using microscopy was widespread in French Guiana. However, owing to the difficulty of maintaining highly skilled microscopists in these remote rural areas and the frequent need for the maintenance of equipment, this gold standard method was replaced by RDT for malaria in some of the health-posts.

RDTs for malaria are specific for one of the three following antigens: Histidine Rich Protein 2 (only produced by *P. falciparum*, PfHRP2), Lactate Dehydrogenase (pLDH) or aldolase. *P. falciparum* secretes three histidine-rich proteins that share repeats of hexapeptides representing the epitopes, and they are recognized by monoclonal antibodies (Mab) raised against PfHRP2 protein. Cross-reactions may therefore occur between the PfHRP2 specific Mab and epitopes of the PfHRP3 protein. These hexapeptides are encoded by the exon 2 part of the gene, which is very polymorphic among isolates [9], [10]. This polymorphism could therefore affect the performances of these RDTs [9], [11]. The pLDH-pan antigen is common to all malaria species but some antigen epitopes are species specific (pLDH-pf for *P. falciparum*; pLDH-pv for *P. vivax*) [12]. The antibody composition of RDTs is thus a key factor in whether the test differentiates the malaria species or not. In French Guiana, the Optimal-IT® kit (pLDH-pan+pLDH-pf) has been used by health posts since the introduction of RDTs in the malaria diagnosis policy in 2000. However, owing to its relatively low sensitivity for the detection of *P. falciparum*, and the recent commercialization of several new kits, the authorities are currently considering switching to the pLDH-pan+PfHRP2 kit. HRP2-based RDTs are indeed more suitable for the diagnosis of *P. falciparum* because of their higher sensitivity, better stability across a wider temperature range and lower cost [3], [13].

Recently, up to 41% of parasites in Peru were reported to lack the *pfhrp2* gene [14]–[16]. These genomic particularities could lead to the misidentification of the species when HRP2 RDTs are used for malaria diagnosis. In French Guiana, this was the case in 2009 for a patient with 0.4% *P. falciparum* parasitemia (sample M618), which was wrongly diagnosed by HRP2 RDTs as “species different from *P. falciparum*” and treated by chloroquine. Owing to the prevalence of chloroquino-resistant parasites (25%) in this region, this could have had serious consequences for the patient [17]. In this context, we therefore evaluated the prevalence of this histidine-rich protein gene family (either exon of the *pfhrp2* gene and *pfhrp3* exon 2) in parasites from French Guiana and its consequences on performances of an HRP2-based RDT, the SD Malaria Ag P.f/Pan® kit.

## Material and Methods

### Sample Collection

**Figure 1:**
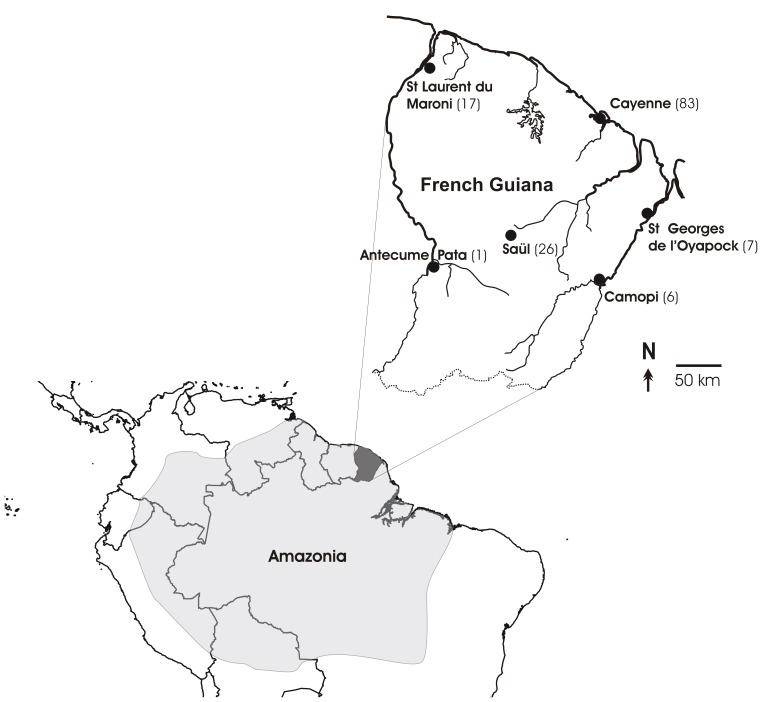
Map of French Guiana, part of Amazonia, South America. Figures in brackets are the number of isolates collected in each health care institution then analysed retrospectively to evaluate the prevalence of *pfhrp2* deletion in French Guiana.

As the National Reference Center (NRC) for Malaria, the parasitology laboratory of the Institut Pasteur de la Guyane is responsible for collecting the majority of malarial samples drawn in French Guiana in order to follow the resistance level of the parasites to antimalarial drugs. In an attempt to evaluate the prevalence of the *pfhrp2* gene deletion, we first performed a retrospective study on a total of 140 *P. falciparum* isolates collected throughout French Guiana in 2009 (Figure 1). Isolates were selected according to this year of collection because this was when the misidentified M618 isolate was reported. Reference strains (genotypically characterized using microsatellite markers) were used as positive and negative controls. These included the undeleted strains 3D7, 7G8 and W2, the *pfhrp2*-deleted strain D10 and the *pfhrp3*-deleted strain HB3 [15], [18].

In a second step, we evaluated the performances of the PfHRP_2_ based RDT, SD Malaria Ag P.f/Pan® (Standard Diagnostics Inc.). For this purpose, we included febrile patients of all ages who consulted for suspected malaria during a prospective study. For a proper assessment of performances (criteria: 95% with a precision of 5% and an alpha error of 0.05), the number of cases required in each of the three groups (negative samples, *P. falciparum* and *P. vivax*) was evaluated at 73, and recruitment continued until at least the required number was reached for each group.

### Regular Malaria Diagnosis

Venous blood for malaria diagnosis was taken from 4 ml samples collected in EDTA tubes.

Reference method. Microscopic examination by an expert microscopist was considered the reference method for malaria diagnosis. Thick and thin blood films were prepared within one hour of blood collection. Thick blood films were stained with Giemsa diluted at 10%, whilst thin blood films were stained using a rapid method (RAL® 555, RAL Diagnostics). Two hundred fields of the thin blood film were examined before classifying the thin smear negative, then 1000 counted white blood cells (WBCs) from the thick smear were observed before classifying the sample as negative. The parasite density estimation was based on an assumed 6,000 WBC/µl of blood.

The microscopic examination results were transmitted to the appropriate physicians within an hour of the blood being drawn. In the case of positive results, patients were treated following the regional guidelines for malaria [19].

Rapid diagnostic tests. The SD Malaria Ag P.f/Pan® (Standard Diagnostics Inc.) detects the presence of pan-pLDH and PfHRP2 antigens. This test was performed according to the manufacturer’s instructions, and the microscopic examination was carried out simultaneously by a second technician. Interpretation of the test results was carried out independently of the microscopic examination and within the time recommended by the manufacturer. Test results were considered invalid if no control band was seen. The tests were guaranteed to have been correctly stored (temperature 24–28°C) and were used within their recommended shelf life.

### Malaria Diagnosis by PCR

In the case of diagnosis discrepancies between RDT and microscopy, a PCR method was used to determine the species present in the sample. DNA was extracted from 200 µl of venous blood collected from EDTA tubes using the QIAmp® DNA mini kit (Qiagen). All the manufacturer’s instructions were followed with the exception of the elution step, which was carried out with 100 µl of elution buffer instead of 200 µl. A nested-polymerase chain reaction (PCR) was performed for samples with non-concordant results. Species-specific nested PCR tests were carried out for each of the four main malaria species, as previously described [20]. The sensitivity of this method in our laboratory was evaluated at 2.5 and 1 parasites/µl of blood to detect *P. falciparum* and *P. vivax*, respectively (data not shown).

### RDT Performance Evaluation

*P. malariae* cases were removed from the study. Mixed *P. falciparum*/*P. vivax* infections were analyzed in the *P. falciparum* group, as the SD Malaria Ag P.f/Pan® only allows the distinction between *P. falciparum* and other species. The performances of the SD Malaria Ag P.f/Pan® kit were expressed by calculating the sensitivity and the specificity for *P. vivax* and *P. falciparum* separately, taking microscopy results as the “gold standard”.

Sensitivity is the ability of the test to detect parasites among samples seen to contain parasites during microscopic examination. The sensitivity value was the number of *P. falciparum* or *P. vivax* samples showing concordant microscopy and RDT results divided by the total number of *P. falciparum* or *P. vivax* results gained through microscopy, respectively.

Specificity is the ability of the test to correctly classify negative samples among negative microscopy results. The specificity value was the number of samples lacking *P. falciparum* or *P. vivax* (i.e. those containing other malaria species+negative samples) divided by the total number of negative *P. falciparum* or *P. vivax* microscopy results, respectively.

Data were analyzed using the Epi-info 3.5.1 software (CDC, Atlanta, GA). Results were accompanied by 95% confidence intervals (95% CI) calculated using the Clopper Pearson exact method [21].

### Detection of the *pfhrp2* and *pfhrp3* Genes

We amplified either exon of the *pfhrp2* gene and the exon 2 of the *pfhrp3* gene from 2 µl of DNA using the primers and conditions described in Table 1. The A262/A263 primer pair amplified the exon1/intron 1 part of the *pfhpr2* gene from 10 bp before the start codon to 305 bp after; the A118/A119 primer pair from 225 bp after the start codon to 274 bp after the stop codon of the *pfhrp2* coding sequence and A266/A261 amplified the exon 2 part of the *pfhrp3* gene from 226 bp after the start codon to 20 bp after the stop codon. PCR products were analyzed by electrophoresis in 1.5% agarose gel. Sensitivities of these PCRs were evaluated at 25 and 50 parasites/µl of whole blood, respectively (data not shown).

**Table 1:** Primer sequences, PCR conditions and expected product sizes.

Gene name	Primername	Primer sequence	[MgCl_2_]	PCR conditions		Product size	Sensitivity (parasite/µl of blood)
*Pfhrp2* exon1/intron1	A262	5′-TTTAATAAAAATGGTTTCCTTC-3′	2 mM	94°C for 15 min; 94°C for 30 sec; 55°C for 30 sec; 72°C for 1 min; 72°C for 10 min	35 cycles	315 bp*	25
	A263	5′-GCTTGAGTTTCGTGTAATAATCT-3′					
*Pfhrp2* exon2	A118	5′-TTCCGCATTTAATAATAACTTGTG-3′	2 mM	94°C for 15 min; 94°C for 30 sec; 55°C for 30 sec; 72°C for 1 min; 72°C for 10 min	35 cycles	1166 bp*	25
	A264	5′-AAAATCGCTATCCCATAAATTACA-3′					
*Pfhrp3* exon2	A266	5′-CCGAATTTAACAATAACTTG-3′	4 mM	As above with an annealing temperature at 60°C		772 bp^**^	50
	A261	5′-TAATCTTCGATTAAATGGATT-3′					

* Based on the HB3 reference sequence (Pubmed accession number: AANS01002100) **Based on the 3D7 reference sequence (Pubmed accession number: NC_004331).

The *pfhrp2* gene was sequenced for a panel of 30 *P. falciparum* isolates identified during the prospective study. These were randomly selected with the exception of M618, the isolate of particular interest. The PCR products obtained after the amplifications, with the primer pairs A118/A264 and A262/A263 were purified using spin columns (QIAGEN) then sequenced using standard dye-terminator sequencing reactions (Applera). The sequences reported in this article have been deposited in the GenBank database (accession number KC558574 to KC558602). Nucleotide sequences were translated to amino acids and each repeat was identified using a previously-described numeric code system [9]. The sequences were aligned using the MegaAlign® software (DNAstar® Lasergene) and categorized into groups and sub groups according to their similarities.

### Ethical Considerations

Analyzed samples were all obtained by blood collections required by the standard medical care for any patient presenting fever on hospital admission in French Guiana. According to the French legislation (article L.1211-2 and related of the French Public Health Code), biobanking and secondary use for scientific purpose of human clinical remaining samples are possible as long as the corresponding patients are informed and has not given any objection to them. In the present research, this requirement is fulfilled: information is given to every patient through the Hospital brochure entitled “Information for patients”, and no immediate or delayed patient opposition was reported by the hospital clinicians to the Malaria NRC.

Moreover, in application of French legislation (article L.1243-3 and related of the French Public Health Code), samples received at the Malaria NRC had been registered for research purpose in the NRC biobank declared to both the French Ministry for Research and a French Ethics Committee which both approved and registered this thematic biobank (declaration number DC-2010-1223; collection N°2). No institutional review board approval is required according to the French legislation.

## Results

### None of the 140 Isolates Lacked the Entire *pfhrp2* Gene

We amplified either exon of the *pfhrp2* gene of 140 *P. falciparum* isolates collected from different parts of the country in 2009 (Figure 1). The M618 case from Saint Laurent du Maroni was included in this genotyping. None of these isolates (95% CI: 0–2.60) contained parasites lacking the *pfhrp2* gene. However, 2.86% (95% CI: 0.78–7.15, n = 4/140) isolates lacked the *pfhrp3* exon 2.

### The SD Malaria Ag P.f/Pan® Kit Demonstrates High Performances in French Guiana

The prospective study to evaluate the performances of the SD Malaria Ag P.f/Pan® kit was conducted from July 2010 to July 2011. During this period, 960 EDTA blood samples were collected at Cayenne Hospital from patients with suspected malaria. The male/female ratio was 1.2∶1 with a median age of 25.8 years [range 1–92 years]. The place of contamination was unknown for most of the patients. According to the data analysis plan, three *P. malariae* cases were excluded and the 7 *P. falciparum/P. vivax* mixed-infections detected by microscopy were analyzed as *P. falciparum* infection. Microscopic examination revealed the majority of the samples (n = 738, 77.9%) to be negative for malaria, whilst 93 (9.7%) and 129 (13.4%) isolates were positive for *P. falciparum* and *P. vivax*, respectively (Table 2). The median parasitaemia values were 0.15% and 0.11% in the *P. falciparum* and *P. vivax* positive samples, respectively. No invalid RDTs (absence of control band) were observed. The SD Malaria Ag P.f/Pan® kit exhibited a sensitivity of 96.8% (95% CI: 90.9–99.3) to detect *P. falciparum* and 86.0% (95% CI: 78.9–91.5) to detect *P. vivax.* The associated specificities were 98.8% (95% CI: 97.9–99.5) and 99.6% (95% CI: 99.0–99.9) for the diagnosis of *P. falciparum* and *P. vivax,* respectively.

**Table 2:** Study of the SD Malaria Ag P.f/pan® performances compared to microscopy as gold standard, French Guiana, July 2010–July 2011.

	Microscopy (n = 28 discrepancies)			
SD Malaria Ag P.f/pan®	*Pf*	*Pv*	Negative	n
***P. falciparum***	**90**	**4**	**6**	**100**
***Other Plasmodium sp.***	**2**	**111**	**1**	**114**
Negative	**1**	**14**	**731**	**746**
**Total**	**93**	**129**	**738**	**960**

Entirely concordant results are shown in the grey fields.

Twenty-eight discrepant results were found between the microscopic examination and RDT results (Table 2). They were all analyzed by PCR (Table 3). The microscopic results were confirmed for 82.1% (n = 23/28) of these cases. In half of the discrepancies, the SD Malaria Ag P.f/Pan® kit had failed to detect *P. vivax* isolates. These false negative results were essentially linked to isolates with a parasite density of less than 0.01% (n = 12/14).

**Table 3:** Diagnosis results after analysis by PCR of the discrepancies observed between the SD Malaria Ag P.f/pan® assay and microscopy, French Guiana, July 2010–July 2011.

		Microscopy results						
		*Pf*		*Pv*		Neg		
PCR results	RDT	*Pv*	Neg	*Pf*	Neg	*Pf*	*Pv*	n
P. falciparum		1		1				1
Other Plasmodium sp.		1		3	14	1		20
Negative			1			5	1	7
**Total**		**2**	**1**	**4**	**14**	**6**	**1**	**28**

Results suggesting the presence of *pfhrp2* and/or *pfhrp3* deleted parasites in the isolates are shown in the grey fields.

Regarding the discrepancies potentially linked to the *pfhrp2* genotype of the parasites, one isolate of interest was identified, namely N500. PCR validation and a second microscopic examination determined that it was a mixed infection (Pf/Pv) missed by both microscopy and the RDT kit. In this isolate, the *pfhrp3* exon 2 was deleted, whilst either exon of the *pfhrp2* gene was present.

As the isolates included in the retrospective study did not contain any parasites lacking either exon of the *pfhrp2* gene, the 93 confirmed *P. falciparum* samples of the prospective study were included in a systematic presence/absence of the entire *pfhrp2* gene and *pfhrp3* exon 2 genotyping processes. Within these 93 isolates, ten DNA were not available for analysis, and two isolates were excluded after PCR confirmed them to be either negative or to contain *P. vivax* (Table 3). None (CI95%: 0–4.45) of the 81 isolates analyzed showed a *pfhrp2* gene deletion. In 7.4% (CI95%: 2.77–15.43, n = 6/81) of cases, a deletion of the exon 2 part of the *pfhrp3* gene was observed.

### Low Polymorphism in the Pfhrp2 Gene of Parasites Circulating in French Guiana

For a better understanding of the PfHRP2 polymorphism displayed by the isolates circulating in French Guiana, 30 samples were genotyped. Twenty-nine *pfhrp2* sequences were obtained and converted into bar code according to their amino acid repeats. Finally, haplotypes were categorized in three groups according to motif similarities (Table 4). The group 1 haplotype prevailed (82.8% *vs* 13.8% and 3.4% for the groups 2 and 3 respectively), and the M618 isolate was part of this group. No polymorphism was observed in the exon 1 part of the *pfhrp2* gene while variability in the microsatellite included in the intron 1 was observed in two samples (16 and 18 repeats versus 15 for the others). Furthermore, no frame shift in the *pfhrp2* sequence of these samples was observed.

**Table 4:** Sequence of *P. falciparum* histidine-rich protein 2 (PfHRP_2_) exon 2 of isolates from French Guiana, 2010.

Group	n	PfHRP_2_ amino acid sequence																																																	
		5′end (from A13to D56)	Motif																																																3′end
1a	14	AVFA……HVDD(Identical to HB3)	1			1	1	1	2	3	2	6	7	7	7					2	2	2	2	2	3	5	5	2	8	2	7							6	7	7	6	7	7			7		10	10	12	CLRH
1b	4			1	1	1	1	1	2											2	2	2	2	2	3	5	5	7	8	2	7							6	7	7	6	7	7	6	6	2	2	10	10		
1c	4					1	1	1	2	3	2	6	7	7	7					2	2	2	2	2	3	5	5	2	8	2	7							6	7	7	6	7	7	6	7	7	10	10	10		
1d	2					1	1	1	2	3	2	6	7	7	7					2	2	2	2	2	3	5	5	2	8	2	7							6	7	7	6	7	7				10	10	10		
2a	3	AVFA……HVDD	1	2	2				2	3	2	6	7				2	2	2	2	2	2	2	2	3	5		7	9	2	7							6	7	7			7	6	2			10	10	12	CLRH
2b	1			1	2				2	3								2	2	2	2	2	2	2	3	5		7	8	2	7	6	2	7	7	6	7	6	7	7				6	6	7	10	6	10		
3	1	AVFA……HVDD	1	1	2				2	3	2	6	7	7	7	7	7	7	2	2	2	2	2	2	3					2	6							6	7	7	6	7	7	6	2					12	CLRH

Repeated sequences were encoded using codes previously published by Baker *et al*., 2005 [9].

## Discussion

The present study is the first report carried out in the eastern part of the Amazonian region to simultaneously assess: i) the genotype of the histidine-rich protein gene family, and ii) the associated performances of an HRP2-based RDT kit. It confirms that these types of RDT are suitable for malaria diagnosis in French Guiana, as none of the parasites in this part of the world lack the whole *pfhrp2* gene and only 4.5% lacked the *pfhrp3* gene.

### High Performances of the PfHrp2-based RDT in French Guiana

In order to be reliable, a method for *P. falciparum* diagnosis should provide a sensitivity of over 95% [22]. The PfHRP2-based kit, SD Malaria Ag P.f/Pan®, demonstrated high performances with sensitivities of 96.8% (95% CI: 90.9–99.3) and 86.0% (95% CI: 78.9–91.5) for the detection of *P. falciparum* and *P. vivax*, respectively. Comparison with previous studies is difficult, as test performances may vary due to i) different geographical variations of parasite populations, ii) levels of disease prevalence and iii) the study design [13], [22]. Nevertheless, the 96.8% sensitivity for the detection of *P. falciparum* correlates with previous results obtained with other PfHRP2 based kits in the region [3], [23]. Sensitivity was lower (86%) in the diagnosis of *P. vivax*. This value is mainly explained by the false negative results obtained for isolates with parasitemia values of under 0.01%. *P. vivax* cases being generally more benign than *P. falciparum*, the SD Malaria Ag P.f/Pan® kit could therefore be considered suitable for malaria diagnosis in French Guiana.

### No Deletion and Limited Polymorphism of the *Pfhrp2* Gene in French Guiana

If we consider the entire set of analyzed samples, these performances were associated with an absence of *pfhrp2* gene deletion (95% CI: 0–1.66, n = 0/221) and 4.5% deletion of the *pfhrp3* exon 2 (CI95%: 2.19–8.16, n = 10/221). This sampling represented 14.8% (221/1495) of the total numbers of *P. falciparum* malaria cases identified in French Guiana on the same period. Even if the majority of the isolates were collected in Cayenne, a non-endemic town, local knowledge on human population movements suggests that malaria samples collected in this town are from different parts of French Guiana. Therefore, these results could be extended to the general parasite population circulating in the region between 2009 and 2011. This absence of *pfhrp2* deletion contrasts with a previous study in the Amazonian region of Peru [15]. The authors reported a deletion prevalence of over 25% for the *pfhrp2* gene and more than 70% for the *pfhrp3* gene. These results, associated with the very high prevalence of *P. vivax* in South America, led WHO to recommend the pLDH-based RDTs in this part of the world. However, results from other parts of the continent are scarce [18], [24]. The results of this study suggest that this deletion phenomenon is uncommon in the western part of South America.

A study was carried out on the genetic variability of this gene, including exon 2 which encodes the epitopes detected by the RDTs. No frame shift and a limited genetic diversity were observed among the 29 analyzed samples. Haplotype 1 was predominant in 82.3% of the isolates. This low level of diversity correlates with the general diversity of *P. falciparum* in French Guiana and is probably linked to the low transmission level of this species within this endemic area [6], [25]. Some repeats described in Africa (repeats 11, 13, and 14), South-East Asia/Madagascar (repeats 13 and 14) were absent in French Guiana [24], [26]. Haplotypes 1d and 3 were previously identified in Brazilian isolates [9]. A correlation between parasitemia and the intensity of the Pf test band was observed (data not shown). The low number of isolates in each group did not allow us to evaluate the correlation between haplotype and RDT sensitivity, and the literature discussing this point is controversial [11], [24], [27].

In our study, the M618 *P. falciparum* isolate was of particular interest as two different PfHRP2-based (SD Malaria Ag P.f/Pan® and Core Malaria®) kits failed to diagnose it, yet it did not lack the whole *pfhrp2* gene, neither the exon 2 part of the *pfhrp3* gene. The possibility of a prozone effect due to an excess of either antigen or antibodies was excluded after testing a 1/10 and 1/100 dilution of the sample (data not shown) [28]. Its *pfhrp2* sequence did not exhibit a frame shift, or particular mutations. Its haplotype was part of the 1 d group composed of two isolates. The second isolate from this group, with a parasitaemia of 0.66%, was detectable by the SD Malaria Ag P.f/Pan®. With a parasitaemia of 0.4%, this failure of detection of the M618 could not therefore be explained by inadequate kit sensitivity. Then, we calculated the product of the number of type 2 × type 7 repeats as previously described to classify the isolates as sensitive or not to detection by a PfHRP2 RDT [9]. With a figure of 72, far above the cut-off point of 43 distinguishing the sensitive isolates, this isolate should therefore be detectable by HRP2-based RDTs. Finally, the absence or a low level of expression of the gene should be considered [29]. However, we were not able to test this hypothesis without parasites adapted to *in vitro* propagation or available RNA extracts.

### PfHrp2-based RDTs are Suitable for Malaria Diagnosis in French Guiana

This study provides important information for laboratories and health policy makers, as it demonstrates that PfHRP2-based RDTs are suitable for malaria diagnosis in French Guiana. At a regional level, human population migration and current knowledge of parasite diversity (homogeneity of the molecular resistant markers, genetic diversity…) lead us to believe that this situation should be the same throughout the Guiana shield [17], [25], [30], [31]. Studies of *pfhrp2* and *pfhrp3* deletions among isolates from other countries located in this region would permit us to verify this assumption. In the absence of such studies and as parasites lacking the *pfhrp2* gene exist on the continent; a regular control of their prevalence is required.
